# Nanoscale Contrast Agents for Ultrasound Imaging of Musculoskeletal System

**DOI:** 10.3390/diagnostics12112582

**Published:** 2022-10-25

**Authors:** Xiaoyi Tang, Mengxin Zhao, Wei Li, Jiaqi Zhao

**Affiliations:** 1 Department of Ultrasound, Changzheng Hospital, Naval Medical University (Second Military Medical University), Shanghai 200003, China; 2 Shanghai Key Lab of Cell Engineering, Department of Nanomedicine, Naval Medical University (Second Military Medical University), Shanghai 200433, China

**Keywords:** musculoskeletal ultrasound, ultrasound imaging, ultrasound contrast agent, nanotechnology, molecular imaging

## Abstract

Musculoskeletal ultrasound (MSKUS) has been recognized as an important method for the evaluation of diseases of the musculoskeletal system, and contrast-enhanced ultrasound (CEUS) technology is becoming an important branch of it. The development of novel materials and tiny nano-formulations has further expanded ultrasound contrast agents (UCAs) into the field of nanotechnology. Over the years, nanoscale contrast agents have been found to play an unexpected role in the integration of precise imaging for diagnosis and treatment of numerous diseases. It has been demonstrated that nanoscale UCAs (nUCAs) have advantages in imaging over conventional contrast agents, including superior biocompatibility, serum stability, and longer lifetime. The potential value of nUCAs in the musculoskeletal system is that they provide more reliable and clinically valuable guidance for the diagnosis, treatment, and follow-up of related diseases. The frontier of advances in nUCAs, their applications, and insights in MSKUS are reviewed in this paper.

## 1. Introduction

Ultrasound imaging, with its inherent advantages of noninvasiveness, convenience, and high safety, is now widely used in clinical practice and has gradually become the first choice for the diagnosis of many diseases [1]. As the use of high-resolution ultrasound (HRUS) in the diagnosis of musculoskeletal diseases became increasingly established, the subspecialty of musculoskeletal ultrasound (MSKUS) [2] emerged. MSKUS is a fast, safe, and cost-effective option for diagnosing and intervening in musculoskeletal disorders, providing real-time dynamic imaging that has advantages over other imaging techniques in sports injuries. Our team conducted a computer-aided, semi-quantitative assessment of microcirculatory perfusion in injured muscle using contrast-enhanced ultrasound (CEUS) [3] that was based on a previous study of textural analysis of skeletal muscle injury, which extended the ultrasound diagnosis of muscle injury to the field of artificial intelligence (AI) and provided innovative ideas for the medical–industrial synergistic development of MSKUS [4].

The development of nanomaterials and technology has revolutionized traditional medicine, providing new options for the diagnosis and treatment of many diseases. Nanoplatforms have the advantages of biological safety, particle size controllability, and blood circulation stability [5]. With these advantages, it can be closely integrated with several disciplines, such as medical molecular imaging, chemistry, physics, biology, and genomics, thereby facilitating the development of many emerging ultrasound contrast agents (UCAs). The widespread use of micron-sized contrast agents has contributed to the explorations of nanoscale UCAs (nUCAs), which can contribute to the CEUS research of musculoskeletal diseases.

This review briefly summarizes the applications of nUCAs in the musculoskeletal system, with the expectation that cutting-edge nanomedicine research and cross-fertilization with nUCAs of the musculoskeletal system establishes a more trustworthy and clinically valuable molecular imaging foundation for the diagnosis, treatment, and follow-up of the associated disorders.

## 2. Distinctive Features of nUCAs and Feasibility of Molecular Imaging in MSKUS

A nUCA is a kind of nanoparticle (NP) that is usually less than one micron in diameter. In contrast, conventional UCAs are micrometers in diameter and are generally used for perfusion evaluation of parenchymatous organs such as the liver [6], but rarely applied to musculoskeletal diseases. The shortcomings of conventional UCAs are that they are limited to imaging within the blood pool and cannot penetrate the vessel wall, thus it is difficult to visualize muscular distribution. In addition, the circulation time of conventional UCAs is relatively short and the instabilities associated with the nanoscale have been overcome in the last decade, attributed to the addition of surfactants significantly improving stability and extending lifetimes [7]. As a result, nUCAs can overcome the shortcomings of conventional micron-sized UCAs, thus enabling traditional CEUS to break through the limitations of blood pool imaging and allow extravascular tissue imaging while enhancing contrast ability [8]. Furthermore, studies have shown that nUCAs have a high safety profile and can be used in patients diagnosed with musculoskeletal diseases combined with renal failure or poor health status with no increase in the incidence of adverse events [9].

**Figure 1:**
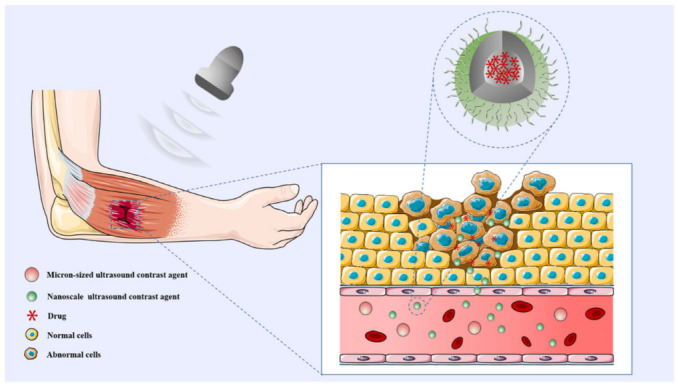
Schematic diagram of the action of nUCAs. The extravascular targeting in the micro-environment of musculoskeletal tissue allows nUCAs to achieve precise targeting and enrichment in damaged or inflamed areas for early diagnosis and assessment. At the same time, it can also serve as a carrier for encapsulated drugs to achieve drug delivery in the target area for rehabilitation, thus realizing the integration of diagnosis and treatment in musculoskeletal diseases.

Due to the enhanced permeability and retention effect (EPR) of nanomaterials, nUCAs accumulate and remain in circulation and tissues for a longer time, thus producing a significant ultrasound-enhanced contrast effect [10]. The incorporation of specific target molecules (e.g., ligands) onto the surface of nUCAs is another effective means of achieving contrast-specific enrichment [11]. As shown in Figure 1, nUCAs with high penetration and specificity can be directly extravasated from the vascular system to the tissue interstitium or muscular cellular surface, where they can be passively enriched by EPR, thus allowing easier and more accurate targeting to certain biomarker molecules of musculoskeletal diseases.

The unique design and fine preparation of nanoplatforms with precise physical, chemical, and biological properties for the treatment of various clinical diseases has now become a research hotspot for novel biological therapies [12,13]. It is worth noting that the ultrasound imaging of stem cells can help show their behavior after transplantation in order to adjust treatment strategies promptly in musculoskeletal systems. Chen et al. reported a novel nUCA that can be used for stem cell imaging and both increased ultrasound imaging contrast to achieve real-time cell tracking/imaging and exhibited excellent adaptability and compatibility [14]. Kubelick et al. injected gold nanospheres-labeled mesenchymal stem cells (MSCs) into animals’ bodies and performed longitudinal cell tracking in dual mode with ultrasound and photoacoustics to continuously track MSCs and detect biological behavior, which provided a real-time monitoring of the stem cell injection at a high spatial and temporal resolution [15]. Moreover, ultrasound-assisted drug delivery has been recognized as a noninvasive method to improve drug penetration and retention, with smaller dose, effective response, and lower systemic toxicity [16]. The ultrasound targeted microbubble destruction (UTMD) technique further enhances the permeability of tissue and cell membranes, thereby overcoming strong biological barriers such as the blood–brain barrier and dense connective tissues [17,18]. In a study conducted by Ho et al., nUCA was a highly efficient nanoplatform that not only enabled enhanced ultrasound imaging but also triggered drug delivery for targeted therapy [19]. Therefore, nUCA provides a carrier for the spatial and temporal modulation of drugs and genes [20], which are expected to be considered as a delivery platform within the musculoskeletal system.

Ultrasound molecular imaging is the construction of targeted acoustic contrast agents by attaching target molecule-specific antibodies or ligands to the surface of the acoustic contrast agents so that the contrast agent actively binds to the target area [21], which is expected to detect musculoskeletal lesions early and achieve accurate “localization, quantification and characterization” diagnosis of lesions. UCAs are the basis of ultrasound molecular imaging, and the development of nUCA undoubtedly provides an excellent opportunity to integrate molecular imaging and diagnosis within the musculoskeletal system. Thus, the distinctive features of nUCAs may contribute to clinical translation in diseases of the musculoskeletal system.

## 3. Applications and Insights of nUCAs of the Musculoskeletal System

Musculoskeletal joints are mostly located in superficial parts of the body and skeletal muscles are rich in blood supply, so injuries caused in daily activities or diseases are quite common in clinical practice. For imaging of the musculoskeletal system, X-ray, computed tomography (CT), and magnetic resonance imaging (MRI) are the main techniques employed as examination procedures. Nanoscale contrast agents, when used in combination with imaging modalities such as CT and MRI, have been studied in preliminary applications in the musculoskeletal system. In a study by Lawson et al., CT nanoscale contrast agents enabled real-time quantitative assessment of articular cartilage, providing a comprehensive image of the disease stage [22]. Xie et al. initially demonstrated a potential translational application of MRI nanoscale contrast agents in cartilage tissue engineering [23]. In addition, Ferumoxytol (Feraheme™) is an FDA-approved iron oxide NP formulation for use as a contrast agent for MRI, as these NPs provide measurable signal changes in labeled stem cells on MRI and thus can be monitored in bone and cartilage repair [24]. However, CT and MRI nanoscale contrast agents still have limitations in terms of timeliness, safety, and economics.

In MSKUS, the color Doppler flow imaging (CDFI) modality, which has been extensively employed in musculoskeletal imaging and sports rehabilitation medicine [2], may evaluate the perfusion characteristics within a lesion area and CEUS can offer a comparatively non-invasive, real-time, dynamic assessment of the microcirculatory perfusion in the musculoskeletal system by showing the activity and metabolic capacity of the tissues, which is essential for the in situ diagnosis of associated diseases [25]. There is growing evidence that nanoscale contrast agents offer unique advantages in the field of diagnosis and treatment of the musculoskeletal system. The representative studies of nanoscale contrast agents related to the musculoskeletal system in the last decade are briefly shown in Table 1. Although the application of nUCAs in the musculoskeletal system is not yet widely studied, it is believed that this technology will be a future research trend.

The diagnostic potential of nUCAs in the microcirculation of the musculoskeletal system has overcome the morphological limitations of conventional imaging modalities, facilitating the acquisition of functional information within deep and superficial tissues and joints. Here, the applications of nUCAs in MSKUS include several diseases, as follows.

### 3.1. Skeletal Muscle Disorders

Muscle disorders are common in the musculoskeletal system. Compared with MRI’s high cost and limitations of static observation pose difficulties for real-time diagnosis, CEUS has the advantage of dynamic imaging with high specificity for the diagnosis of injury in soft tissues, such as muscles.

Inflammatory changes in muscle contusions are mainly characterized by the production of reactive oxygen species (ROS). Using H_2_O_2_ as a biomarker, Kim et al. explored the feasibility of poly(vanillyl alcohol-co-oxalate) (PVAX) NPs and poly(vanillin-co-oxalate) (PVO) NPs as contrast and therapeutic agents, respectively, for muscle contusions. These nanopolymers react rapidly with H_2_O_2_ to produce carbon dioxide bubbles at the injury site, thus enhancing the ultrasound imaging contrast at that site. Meanwhile, they also inhibit the expression of pro-inflammatory cytokines and the infiltration of inflammatory cells. Animal experiments also verified their anti-inflammatory and anti-apoptotic effects, with translational potential for clinical application [28,29].

Skeletal muscle ischemia–reperfusion injury is a dramatic inflammatory response caused by a short period of recovery from ischemic supply of skeletal muscle blood flow from various causes. It is considered one of the pathophysiological mechanisms of peripheral vascular diseases, such as diabetic microangiopathy. Similar to muscle contusion, the production of high levels of H_2_O_2_ is a major cause of ischemia–reperfusion injury. Lee et al. reported an H_2_O_2_-activatable multifunctional hydroxy benzyl alcohol-incorporated copolyoxalate (HPOX) NP. The investigators used a mouse hindlimb ischemia–reperfusion injury model to image H_2_O_2_ in vivo, while using it as a drug delivery carrier for in vivo antioxidant and anti-inflammatory therapy, showing preliminary potential for clinical applications [30]. Later, Jung et al. used their prepared curcumin-loaded PVAX NPs to assess the intensity of the inflammatory response and therapeutic effects in mice with ischemic injury. PVAX NPs loaded with curcumin were shown to exhibit stronger antioxidant and anti-inflammatory activities than the blank control group [27]. In addition, Negishi et al. evaluated the feasibility and efficacy of nUCAs for phosphorodiamidate morpholino oligomer (PMO) delivery in dystrophic mdx mice and suggested that the combination of bubble liposomes and ultrasound exposure may enhance PMO delivery for the treatment of Duchenne muscular dystrophy (DMD) [26].

Although the application of CEUS examination with UCAs on skeletal muscle has emerged recently, CEUS has shown the potential to be a useful tool for monitoring the extent of muscle involvement, the progress of regenerative processes, and even the effectiveness of anti-inflammatory treatments. Therefore, the exploration and application of nUCAs will bring a new approach to the diagnosis and treatment of muscle disorders.

### 3.2. Arthritic Diseases

Globally, the prevalence of arthritic illnesses has remained high in recent years. It is important to note that chronic nontraumatic arthritic illnesses are more prevalent, primarily rheumatoid arthritis (RA) and osteoarthritis (OA), which, in severe cases, can cause disability and functional impairment [35].

Synovitis is the most important pathological abnormality of RA [36]. Ultrasound is more sensitive and specific than other clinical examinations in detecting synovial inflammation. Some studies have shown that CEUS can explore synovial inflammatory lesions and quantitatively/semi-quantitatively evaluate activity and perform molecular imaging of arthritis [37]. Zhao et al. targeted inflammatory biomarkers of RA to vascular endothelial growth factor (VEGF) for early diagnosis and accurate assessment of synovial vascular inflammatory lesions by Ultrasound microbubble-targeted imaging [38]. There are also many scholars focusing on therapeutic applications. Tang et al. reported multifunctional nUCAs that effectively carried Indocyanine green (ICG) and O_2_. The results showed that the ICG-mediated sonodynamic effect produced significant cytotoxic effects on synovial fibroblasts, leading to noninvasive treatment [31]. Gong et al. used nUCAs loaded with MSCs to treat RA. The nUCAs can be used for intracellular labeling and tracking of MSCs. In the study, nUCAs loaded with MSCs were transplanted into arthritic rats and ultrasound imaging allowed real-time tracking of MSC migration and homing. Treatment with the combination of nUCAs loaded with MSCs and methotrexate (MTX) resulted in greater immunosuppression and bone/cartilage regeneration compared to other forms of treatment [33].

New prospects for the treatment of arthritis are also made possible by advances in optical imaging technologies. Among them, ICG-enhanced fluorescence imaging has been established and employed for the diagnosis of RA in animal models. Wu et al. further combined ultrasound drug-controlled release therapy with a phospholipid-based nanoscale drug delivery system to develop MTX-loaded and ICG-targeted acoustic liposomal multifunctional contrast agents. With the aid of near-infrared fluorescence imaging, the entire targeting process was visualized with low-intensity ultrasound at fixed-points to realize controlled release of MTX [32]. In addition, in a study carried out by Chen and collaborators, endogenous melanin contrast agents encapsulated by poly-L-lysine acted as positively charged contrast agents. Accurate photoacoustic imaging (PAI) of cartilage degeneration, through interaction with anionic glycosaminoglycan (GAG) in cartilage, enabled accurate detection and assessment of cartilage degeneration in OA at an early stage [34].

These findings lay a foundation for the early detection and focused therapy of synovial inflammatory lesions in RA and other inflammatory diseases. It is anticipated that the rapid advancement of nUCAs will open up new possibilities for the use of integrated research in the diagnosis and treatment of arthritis through multimodal imaging, using a combination of optical and acoustic contrast agents.

### 3.3. Bone Regeneration and Repair

In recent years, vascularization has emerged as an effective strategy for bone regeneration [39]. The use of CEUS in bone defects and fracture healing has been gradually evaluated and accepted. Compared to color Doppler ultrasound or energy Doppler ultrasound, CEUS enables earlier real-time monitoring of epiphyseal and intraosseous blood flow reconstruction that occurs during healing [40]. Many studies have tried to deliver some of the factors that contribute to osteogenesis, including bone morphogenetic protein (BMP), VEGF, and MSCs [39,41].

Advances in nano-biomaterials also offer promising opportunities for bone regeneration therapy. The synthetic polymer poly(lactic-co-glycolic acid) (PLGA) is a biodegradable material with useful properties. Jin et al. combined low intensity pulsed ultrasound with polymer PLGA nanosystems and found the synergistic controlled release of growth factors achieved both stable growth and early differentiation of osteoblasts in bone regeneration therapy [42]. Bari et al. synthesized mesoporous bioactive glasses containing Cu in the form of spherical nanosystems. The organic combination of multiple elements allowed the nanosystem to exhibit excellent antimicrobial capabilities for the prevention of infections associated with bone defects and was expected to serve as a multifunctional therapeutic agent for bone regeneration [43].

Ultrasound-mediated targeted therapy is considered a noninvasive and effective method. Gong et al. prepared a type of PLGA UCAs loaded with VEGF. Validated by experiments in rats, the results showed that UTMD mediated the release of VEGF-coated UCAs from local bone defects, which significantly promoted osteogenic expression in vitro and promoted bone regeneration and repair in vivo [44]. Nimikou et al. reported an ultrasound-responsive gene-activated matrix containing polymeric UCAs, BMPs, fibronectin/collagen hydrogel matrix, and C2C12 cells and demonstrated that it had the ability to achieve osteogenic differentiation in vitro [45].

Overall, UCA is a promising approach with biological capabilities in bone repair, while the application of nanomaterials can give new hope to bone regeneration therapy. These studies provide new insights, suggesting that novel nUCAs may combine the above-mentioned advantages to become a new option for noninvasive visualization of treatments in bone tissue engineering and regenerative medicine in the future.

### 3.4. Other Musculoskeletal Disorders to Be Explored for Application

In addition to the studies mentioned above, other related disorders in the musculoskeletal system are worthy of attention. Ultrasound-activatable nanoscale catalysts have shown excellent antimicrobial properties in osteomyelitis, providing a viable strategy for the in situ treatment of osteomyelitis [46]. CEUS has been reported to be more sensitive in showing intra- and peritendinous vessels, not only to assess their dynamic perfusion, but also to show the blood flow in the periosteum, bone, and adjacent soft tissues with higher contrast [47]. As a potential parameter of tissue viability, CEUS can reflect the functional properties of tendons. Moreover, studies have been conducted to explore in vivo gene transfection by UCAs in tendons in an attempt to use long-term ultrasound-assisted expression of genes for tendon repair [48]. Similarly, CEUS can visualize muscle perfusion after rotator cuff repair for impaired muscle perfusion and be used as a quantitative method to assess rotator cuff injuries [49].

Interestingly, CEUS evaluation is advantageous to the initial diagnosis, monitoring the effectiveness of therapeutic interventions, and the evaluation of the perfusion status of the hip joints after surgery in pediatric inflammatory joint diseases [50]. In addition, quantitative CEUS allows precise assessment of microcirculatory perfusion, as a surrogate parameter for tissue viability and metabolism, and objectively monitoring the progression of postoperative microcirculatory changes. Furthermore, CEUS is also expected to be an additional diagnostic tool for predicting the malignant potential of bone tumors, due to varying enhancement patterns [51]. Hence, CEUS not only allows for early identification and initial treatment of patients with related injuries but is also expected to be used to monitor the process of blood flow reconstruction, thus facilitating the postoperative rehabilitation of patients.

For clinical applications in the quantitative evaluation of CEUS, or the bio-diagnosis and treatment of the musculoskeletal system, the development of nUCAs that are distinct from ordinary UCAs holds tremendous promise. To accomplish non-invasive applications and to maximize clinical advantages in youngsters, the elderly, and patients in poor health, it may be necessary to develop a safer and more effective nano-platform for the musculoskeletal system.

## 4. Summary and Perspectives

This paper reviewed the applications and insights of nUCAs in the musculoskeletal system. nUCAs have contributed significantly to the development of targeted molecular imaging technology; the introduction of nanotechnology has promoted the development of a new generation of integrated multifunctional molecular imaging contrast agents for musculoskeletal imaging-guided visualization and treatment.

Although nUCAs have shown potential for applications in the treatment of the musculoskeletal system, there are still several critical issues that should not be overlooked. In terms of material preparation, with the increasing maturity of tiny nano-formulations and the diversification of nanoplatforms, it is worth considering how to obtain a balanced combination between nanomaterials and ultrasound imaging capabilities. In terms of basic research, nUCA needs to be combined with specific target molecules in the field of ultrasound molecular imaging. Unlike solid tumors, for example, where specific high expression of certain molecules is apparent, finding the right target molecule in musculoskeletal diseases is a key issue. Last but not least, in terms of clinical translation, nanoscale contrast agents have not been formally approved for clinical use. Only BR55 [52] is currently in preclinical trials and the application of nanocarriers still lacks large sample studies to assess biosafety and reliable biocompatibility. Many nUCAs under study are primed for bimodal or even multimodal imaging and combination with modalities such as fluorescence imaging and PAI can help enhance the contrast and resolution of in vivo imaging [53,54]; however, the expected imaging and therapeutic functions of multimodal contrast agents in the human body are yet to be realized. Therefore, exploring and refining the new ultrasonic nanotechnology and studying its biosafety issues will accelerate the translation of some nUCAs for clinical applications.

The rise of next-generation AI technology has led to a new stage in the rapid application of ultrasound for the diagnosis and treatment of the musculoskeletal system. The future development of medicine, and engineering integration with new multimodal nanoscale ultrasound contrast imaging, will further expand the application prospects of nUCAs in the diagnosis and treatment of the musculoskeletal system and demonstrate its powerful practical value.

## Figures and Tables

**Table 1:** A brief summary of the typical nanoscale contrast agents related to musculoskeletal system in the last decade.

Year	Modality	Diameter	Disease	Application	Ref
2014	US	400–500 nm	Duchenne muscular dystrophy	Imaging and gene delivery	[26]
2018	US	~360 nm	Muscle of peripheral artery disease	Imaging and drug delivery	[27]
2017	US	~400 nm	Musculoskeletal contusion injury	Imaging and anti-inflammatory therapy	[28]
2021	US+FOI	~330 nm	Musculoskeletal contusion injury	Imaging, diagnosis and simultaneous treatment	[29]
2013	BLI	~450 nm	Muscle ischemia reperfusion injury	Imaging and drug delivery	[30]
2017	US+PAI	~278 nm	Rheumatoid arthritis	Imaging, photodynamic therapy and sonodynamic therapy	[31]
2020	FOI	~113 nm	Rheumatoid arthritis	Imaging and drug delivery	[32]
2022	US	~218 nm	Rheumatoid arthritis	Imaging and stem cell tracking	[33]
2018	PAI	~39 nm	Osteoarthritis	Imaging	[34]
2019	MRI	17 nm	Osteoarthritis	Imaging	[23]
2021	CT	3–6nm	Osteoarthritis	Imaging	[22]
2019	MRI	~6 nm	Degenerative bone diseases	Imaging and stem cell tracking	[24]

Abbreviations. Ref: Reference; US: Ultrasound; FOI: Fluorescent Optical Imaging; BLI: Bioluminescence imaging; PAI: Photoacoustic Imaging; MRI: Magnetic Resonance Imaging; CT: Computed tomography.
